# Autism, oxytocin and interoception

**DOI:** 10.1016/j.neubiorev.2014.09.012

**Published:** 2014-11

**Authors:** E. Quattrocki, Karl Friston

**Affiliations:** The Wellcome Trust Centre for Neuroimaging, UCL, 12 Queen Square, London WC1N 3BG, UK

**Keywords:** Autism, Oxytocin, Interoception, Bayesian predictive coding, Neuromodulation, Active inference, Emotional affordance, Sensory attenuation, Self-awareness

## Abstract

Autism is a pervasive developmental disorder characterized by profound social and verbal communication deficits, stereotypical motor behaviors, restricted interests, and cognitive abnormalities. Autism affects approximately 1% of children in developing countries. Given this prevalence, identifying risk factors and therapeutic interventions are pressing objectives—objectives that rest on neurobiologically grounded and psychologically informed theories about the underlying pathophysiology. In this article, we review the evidence that autism could result from a dysfunctional oxytocin system early in life. As a mediator of successful procreation, not only in the reproductive system, but also in the brain, oxytocin plays a crucial role in sculpting socio-sexual behavior. Formulated within a (Bayesian) predictive coding framework, we propose that oxytocin encodes the saliency or precision of interoceptive signals and enables the neuronal plasticity necessary for acquiring a generative model of the emotional and social ‘self.’ An aberrant oxytocin system in infancy could therefore help explain the marked deficits in language and social communication – as well as the sensory, autonomic, motor, behavioral, and cognitive abnormalities – seen in autism.

## Highlights

• We review the role of neuromodulation in interoception and emotional inference.
• We consider the known neuromodulatory roles of oxytocin pertinent to interoception.
• We propose a model for impaired interoception giving rise to the autistic phenotype.
• We review how findings from the autistic literature support this model.
• We describe how this analysis could suggest therapeutic strategies.

## 1 Introduction

This review presents cellular, anatomic, physiologic, pharmacologic, genetic and behavioral evidence that speaks to a failure of the oxytocin system early in development. Using recent advances in our understanding of learning and inference, we consider how this single but pervasive deficit could result in the autistic phenotype.

Although structural brain abnormalities (Greimel et al., 2013), soft neurological signs (Tani et al., 2006), seizures (Amiet et al., 2008), motor disturbances (Rogers, 2007) and autonomic dysregulation (Benevides and Lane, 2013, Kushki et al., 2013) commonly occur in autism, it remains a clinical diagnosis with an elusive neurobiological etiology. Kanner (1943) first described autism in 1943 as a relatively rare condition; yet, in 2012, nearly 1 in 50 children between the ages of 6–17 carried a diagnosis of autism (National Center for Health Statistics, 2013). Numerous theories have been proposed to explain autism. Neurobiologically based theories include: autonomic dysregulation (Hutt et al., 1964, Porges, 1995); mirror neuron system deficits (Williams et al., 2001); neuronal migration abnormalities (Bailey, 1998); an imbalance of excitatory and inhibitory neurons (Rubenstein and Merzenich, 2003); and dysfunctional connectivity with sparse long range connections but excessive local connectivity (Belmonte et al., 2004). Cognitive theories include: deficits in Theory of Mind (Baron-Cohen et al., 1985); executive function and imitation difficulties (Rogers et al., 1996); weak central coherence (Frith and Happe, 1994); complex processing deficits (Minshew et al., 1997); and attentional deficits (in orienting, disengaging, and switching) (Courchesne et al., 1994). Theories focused on the social and behavioral symptoms include: dysfunctional social and affective relations (Hobson, 1991); impaired joint social attention (Mundy et al., 1990); reduced social motivation (Dawson et al., 1998); empathizing vs. systemizing (Baron-Cohen, 2006); and, what has been characterized as the extreme male brain (Baron-Cohen, 2002).

Recently, a Bayesian model for autism has been advanced, suggesting that “people with autism see the world more accurately – as it really is – as a consequence of being less biased by prior experiences.” (Pellicano and Burr, 2012). This formulation of weak predictions and excessive sensitivity to sensory stimulation can account for the nonsocial symptoms of autism; including the hypersensitivity to environmental stimuli, savant qualities, and their reduced susceptibility to visual illusions. However, it does not provide a complete explanation for the social and communication deficits characteristic of autism, nor does it explain why autistic children fail to develop precise prior beliefs or appropriate models of their social and emotional world.

In this paper, we extend the Bayesian model to incorporate neurodevelopment and its physiologic substrates by proposing that a dysfunction in the oxytocin system, early in life, could account for the development of autism. As a key mediator of birth (Dale, 1909), lactation (Hatton and Wang, 2008, Nickerson et al., 1954), the suckling response (Schaller et al., 2010, Wrobel et al., 2010), pair bonding (Insel et al., 1995), maternal care (Francis et al., 2000) and affiliative behaviors (Baumgartner et al., 2008, Kirsch et al., 2005, Kosfeld et al., 2005, Zak et al., 2007), oxytocin stands as the most likely candidate to orchestrate the emergence of the social and emotional brain (Adolphs, 2009). In light of this, a Bayesian formulation of the function of oxytocin may provide a formal framework for understanding aberrant social inference and learning in autism and potentially suggest therapeutic strategies.

In brief, we suppose that an early pathophysiology in the oxytocin system could disrupt the assimilation of interoceptive signals and exteroceptive cues within generative models of the ‘self’. These primary deficits would impair the child's ability to assign salience to socially relevant signals in the environment and disrupt the sensory attenuation necessary for proper homeostatic regulation, coordinated movement, and an outward focus during social encounters—thus undermining the imitation-based observational learning that normal children enjoy. Without a predilection for social stimuli and imitative responses, the behavioral repertoires or routines that eventually progress – through hierarchical assimilation into social interactions, language, biological movement, Theory of Mind and empathic responses – would fail to develop.

We start with a brief overview of the Bayesian brain and how oxytocin might implement the neuromodulation necessary for successful (interoceptive) inference and learning. With this theoretical perspective in place, we then consider the known neuromodulatory function of oxytocin and how aberrant neuromodulation might give rise to the autistic phenotype. Finally, we review the empirical evidence supporting this theory—evidence that speaks to abnormal interoceptive processing and subsequent failures of socio-emotional learning in autism and suggest how this theory might inform our approach to therapeutic treatment.

## 2 The Bayesian brain, neuromodulation, and interoception

### 2.1 Predictive coding

**Fig. 1:**
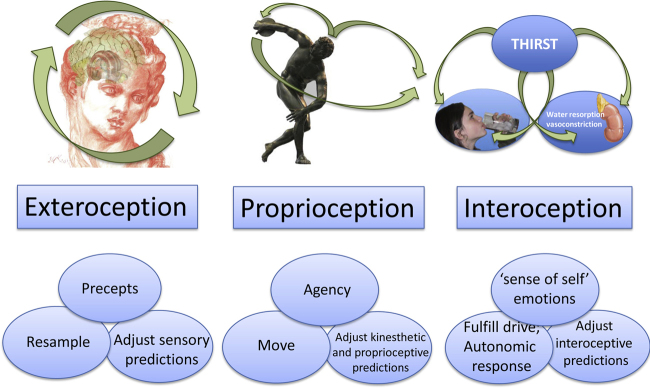
*Predictive coding.* This schematic introduces predictive coding in terms of exteroceptive, proprioception and interoception: Exteroception uses primary senses such as sound, light, and discriminatory touch to build models or precepts of the external world. Proprioception processes proprioceptive and kinesthetic information from the body to allow movement and provide a sense of agency. Interoception allows models of the internal “self” to be constructed and emotional feelings to be inferred through visceral sensations such as temperature, stretch and pain from the gut, light (sensual) nondiscriminatory touch, itch, tickle, hunger, nausea, thirst, sleepiness, and sexual desire. **Left panel**: This illustration portrays the brain as an inference machine, using predictive coding to form perceptual representations (inferences) of the environment from light signals by extracting inherent structure from visual sensory information. In this predictive coding, visual signals are transmitted up the hierarchical processing pathway from the eyes (via the retina, optic nerve, superior colliculi, and lateral geniculate) first to primary visual sensory cortex and then to higher cortical regions that instantiate perceptual inference. The curved arrows in the figure symbolize the iterative and recursive nature of predictive coding. This illustration simplifies the hypothesis testing—that would normally occur with reference to generative models at multiple levels in the central nervous system into one loop. In the brain, neuronally encoded expectations or explanations for sensory input generate predictions that are transmitted to lower layers and ultimately sensory cortex. In hierarchical predictive coding, each level is trying to predict the level below by sending top-down predictions to form prediction errors that are then returned to improve the higher predictions. The implicit comparison of top-down predictions and bottom-up evidence allows for ascending transmission of only the relevant information (prediction error) that violates the top down predictions. These violations (prediction errors) travel up the hierarchy to inform higher cortical levels. The expected precision (inverse variance) of the prediction errors determines the degree to which these signals influence representations at higher levels. **Middle panel**: This depiction of embodied (active) inference, illustrates how action itself becomes the fulfillment of a forward model (prediction). The teal arrow represents the signal attenuation, in the form of corollary discharge that occurs when movements, or behaviors, are self-generated. In the case of movement, motoric predictions constitute descending signals that suppress ascending proprioceptive prediction errors that would otherwise prevent the fulfillment of the prediction. Successful attenuation, and therefore movement, largely depends upon the proper construction and utilization of predictions about the physical ‘self’. **Right panel**: The equivalent action in the interoceptive domain is the fulfillment of an internal prediction or drive, either by adjusting the autonomic response, or by initiating a behavior. The interoceptive equivalent to throwing a disc – as depicted in the middle panel – might be fulfilling the prediction of thirst. Prediction errors will drive either an autonomic reaction that will deploy a physiological response such as vasoconstriction or water resorption in the kidney, while at the same time boosting the affordance of water and prescribe the behavior that will lead to drinking, thus fulfilling top-down predictions. Because these actions are self-generated, they require sensory attenuation that might otherwise subvert the execution of either the behavior or the homeostatic adjustment. Our focus in this paper is on the attenuation of interoceptive prediction errors—or interoceptive attenuation. (For interpretation of the references to color in this figure legend, the reader is referred to the web version of this article.)

Current concepts of cerebral information processing suggest the brain constructs probabilistic internal models of the world which are continually updated to efficiently explain the causes of its sensory inputs (Dayan et al., 1995, Friston, 2005, Gregory, 1968, Gregory, 1980, Helmholtz, 1885). Predictive coding is generally regarded as the most neurobiologically feasible scheme for updating and refining these internal models (Mumford, 1992, Rao and Ballard, 1999, Srinivasan et al., 1982). Predictive coding forms perceptual representations by testing (top-down) hypotheses or predictions against (bottom-up) sensory evidence. By applying iterative hypothesis testing, the brain can incorporate new sensory evidence using the discrepancy between the observed and predicted sensory input (Friston, 2005). This discrepancy between model based predictions and sensory input is known as *prediction error*. In predictive coding schemes, prediction errors are transmitted from sensory cortex up hierarchical processing pathways to update representations in higher cortical levels. These representations correspond to probabilistic expectations (encoded by neuronal activity) about the causes of sensory input and adjust top-down predictions to reduce prediction errors at lower levels. The implicit comparison of descending (top-down) predictions and ascending (bottom-up) evidence selects the relevant information (prediction errors) that violate top down predictions. These violations inform higher cortical levels—serving to recursively update the predictions such that optimal representations emerge at each level of the cortical hierarchy, thereby furnishing an explanation of the sensory world at multiple levels of description (see Fig. 1). In this hierarchical predictive coding or inference framework, high level expectations are referred to as (empirical) priors or prior beliefs.

Neuromodulatory mechanisms participate in predictive coding schemes at every stage of the process, from controlling relative gain of individual signals, to implementing the learning associated with generative models or prior beliefs, and deploying the predictive models necessary for action to occur. Neuromodulatory mechanisms control the recursive message passing – implied by predictive coding – by altering the relative influence of ascending prediction errors and descending predictions. This balances the confidence placed in prior beliefs, relative to the sensory evidence for those beliefs. The confidence placed in sensory evidence depends on the context dependent assignment of expected precision at different hierarchical levels and plays a key role in evidence accumulation, through attentional selection, and attenuating unhelpful or predictable sensations during active exchanges with the world (Feldman and Friston, 2010, Friston and Kiebel, 2009, Shipp et al., 2013, Vossel et al., 2014).

The contribution of various neurotransmitters in neuronal implementations of predictive coding has been considered in depth elsewhere (Corlett et al., 2009, Friston and Kiebel, 2009, Friston et al., 2012, Hirayama et al., 2004, Yu and Dayan, 2005). However, the role of neuropeptides, and in particular, oxytocin, has not been explored within the predictive coding framework. In contrast to traditional neurotransmitters, studies of simple organisms suggest that neuropeptides, although often only transiently deployed, can influence complex behavior over prolonged periods of time (Bernheim and Mayeri, 1995, Knox et al., 1992, Kupfermann, 1970, Levitan et al., 1987, Quattrocki et al., 1994), for review see (Harris-Warrick, 2011). Neuropeptides accomplish these protracted influences by promoting cross modal integration (DeLong and Nusbaum, 2010, Macosko et al., 2009), by altering the sensitivity of microcircuits to sensory inputs (Nusbaum and Blitz, 2012) and by providing organizational coherence to central pattern generators (Garrison et al., 2012). In higher species, neuropeptides play a similar neuromodulatory role to choreograph the acquisition, consolidation, and implementation of instinctual behaviors and drives (Potts, 2006, Viviani et al., 2011) and to facilitate cross modal integration (Lee et al., 2013). In short, these highly conserved molecules are strategically placed at the heart of neuronal processing, for review see (Dickinson, 2006).

### 2.2 Oxytocin and interoception

Oxytocin is a phylogenetically ancient and ubiquitous peptide that mediates many aspects of reproduction, including copulation, parturition, lactation, and more complex behaviors related to care giving and pair bonding. The paraventricular and supraoptic nuclei of the hypothalamus synthesize oxytocin and deploy it via the posterior pituitary to act as a hormone on peripheral organs and via direct projections and passive diffusion in the central nervous system to modulate neuronal activity—through interactions with the oxytocin receptor (de Wied et al., 1993). Oxytocin, together with vasopressin and acetylcholine, regulate many homeostatic bodily functions associated with the parasympathetic arm of the autonomic nervous system (Ropper and Samuels, 2009). In the central nervous system, oxytocin plays a complementary role to ensure reproductive success by augmenting prosocial and affiliative behaviors. Thus, if predictive coding instantiates the information processing in the brain that underwrites reproductive success, oxytocin may participate in the neuromodulation necessary to elicit the emotions, behaviors, and cognition that are crucial to accomplishing prosocial and affiliative objectives.

Fundamental to maintaining homeostasis and implementing behaviors to satisfy instinctual operations are the perceptual inferences provided from interoception. Interoception, the process of perceiving one's own internal state, considers sensations such as stretch and pain from the gut, light (sensual) nondiscriminatory touch, itch, tickle, temperature, hunger, nausea, thirst, sleepiness, and sexual desire. These sensations are used to maintain homeostasis, to infer emotional states, to elicit behaviors, and ultimately to provide a sense of the embodied self (Craig, 2002). Predictive coding schemes have been enlisted to explain perceptual inference in exteroception (Rao and Ballard, 1999) and proprioception (Adams et al., 2013). More recently, predictive coding has also been applied to interoception (Seth et al., 2011). Interoceptive prediction errors ascend autonomic pathways; inform limbic regions and pervade the cortical hierarchy—where information about the internal milieu converges with exteroceptive and proprioceptive signals. The assimilation of interoceptive signals into higher-level representations of our sentient ‘self’ most likely occurs in the insular, cingulate, and ventromedial prefrontal cortices (Apps and Tsakiris, 2014, Damasio and Carvalho, 2013). The expectations from these higher level representations then furnish the basis for top-down predictions – that endow perceptual inferences with an affective valence – literally ‘gut’ feelings – that are thought to be a key component of emotional salience and self-awareness (Critchley and Seth, 2012, Seth, 2013, Seth et al., 2011). See Fig. 1 (right panel).

Oxytocin influences the transmission of interoceptive signals, providing information about the internal milieu pertinent to homeostasis and reproduction. The participation of oxytocin in interoceptive signal transmission has been associated with almost every modality, including: olfaction, for review see (Wacker and Ludwig, 2012); taste (Popik and Van Ree, 1993); light (sensual) touch, for review see (Ishak et al., 2011); sensations of warmth (Uvnas-Moberg, 1997); thirst (James et al., 1995); sexual desire, for review see (Bancroft, 2005); as well as appetite, for review see (Leng et al., 2008); pain, for review see (Rash et al., 2014); and thermoregulation, for review see (Chaves et al., 2013). Oxytocin has even been associated with the interoceptive signals of itch (Yaksh et al., 2014), sleepiness (Voloschin and Tramezzani, 1979), and nausea (Seng et al., 2013).

Oxytocin receptor distribution in the brain supports a role for oxytocin in the transmission and modulation of interoceptive signals (prediction errors) in neuronal circuits. Oxytocin receptors are found in key hubs of the autonomic nervous system (the solitary nucleus of the brainstem (NTS) and the hypothalamus), they populate limbic areas (the bed nucleus of the stria terminalis and the central and basolateral nuclei of the amygdala), and inhabit the major source of acetylcholine in the brain (the nucleus basalis of Meynert) (Boccia et al., 2013, Loup et al., 1991). Although direct binding studies have not specifically identified oxytocin receptors in higher cortical areas, human neuroimaging and animal electrophysiology studies suggest that oxytocin has specific and targeted effects on the medial prefrontal cortex (Ninan, 2011, Qi et al., 2009, Qi et al., 2012, Smeltzer et al., 2006), the insula (Lawson et al., 2012, Riem et al., 2011, Striepens et al., 2012), the anterior cingulate and the inferior frontal gyrus (Riem et al., 2011). Thus, the functional anatomy of oxytocin strategically situates this neuropeptide system in synaptic locations involved in the interoceptive pathways that mediate emotions, sexual and social drives, social learning and memory as well as areas pertinent to empathy and theory of mind.

### 2.3 Oxytocin and inference

The physiology of predictive coding requires flexible neuronal communication on a short time scale (i.e., neuromodulation of synaptic gain or efficacy) for perceptual inference and the more enduring changes in synaptic plasticity that ensue as the learning of generative models develop. The neural mechanisms subserving gain or precision control (during sensory integration and attentional selection) have not been conclusively determined, but several mechanisms hold explanatory promise. Among these, communication through changes in synchronous activity (coherence) has the adaptive power to account for many of these operations on a time scale relevant to perceptual inference (Fries, 2005). These models assert that synchronous activity among neuronal populations provides a ubiquitous form of gain control and selection of processing channels. The emergence of coherent gamma oscillations within an assembly of cortical neurons may underlie the binding of sensory integration (Engel et al., 2012, for review see Sedley and Cunningham, 2013). A pervasive effect of oxytocin, in both the body and the central nervous system, is to initiate synchronized neuronal activity (Rossoni et al., 2008). For instance, oxytocin coordinates the simultaneous contraction of uterine smooth muscles, thus allowing for more forceful labor (Fuchs and Fuchs, 1984). In providing positive feedback for its own release in the hypothalamus, it recruits neurons to fire in synchronous bursts (Hatton and Wang, 2008). Furthermore, oxytocin facilitates synchronous and coordinated activity during lactation (Hatton and Wang, 2008). In short, the idea that oxytocin contributes to a coordinated neuronal response, is well supported in the body and hypothalamus, for review see (Bealer et al., 2010). Although conjecture, it is not a large leap to speculate that oxytocin might also facilitate the same type of synchronous firing in the cortex, thus allowing this neuropeptide to influence the neuronal message passing that underlies hierarchical inference in the brain.

### 2.4 Neuromodulation and precision

Neuromodulatory effects of the sort associated with oxytocin (described below) play an important and ubiquitous role in predictive coding. The relative precision of prediction errors at different levels of the hierarchy, or in different sensory modalities, has a profound effect on the recurrent hierarchical message passing that underlies predictive coding: the potency of ascending prediction errors on subsequent hierarchical processing depends upon their precision (Friston, 2005). Computationally, precision is inverse variability or uncertainty. Thus, precision encodes the confidence in the information reported by prediction errors. Physiologically, precision represents the postsynaptic gain control or baseline modulation of neuronal populations reporting prediction errors (Mumford, 1992, Shipp et al., 2013). This simple mechanism can account for enhanced attention or attentional biases of the sort seen in the Posner paradigm and its electrophysiological correlates – such as biased competition (Feldman and Friston, 2010), when specific stimuli, such as eye gaze, captivate processing resources – to the exclusion of other stimuli in the same scene or receptive field. Heuristically, ascending prediction errors from sensory cortex are the newsworthy (unpredicted) component of sensory information, while precision basically adjusts the gain or ‘volume’ of different sensory channels competing for influence on higher level representations.

In the setting of interoceptive inference, precision adjusts the gain of signals conveying homoeostatic and reproductive (or socially) relevant information. Early in development, interoceptive precision will allow salient interoceptive information, such as satiety, temperature, thirst, pain, tickle, smell and taste signals to be transmitted up hierarchical (interoceptive) pathways to progressively more multimodal areas to allow for accurate inferences. Learning, in the form of strengthened synapses or a more defined network in higher cortical areas, depends upon these ascending (precision weighted) prediction errors. Later, prediction errors that have successfully been assimilated into the higher level generative models through learning will influence the predicted precision of signals coming from specific interoceptive channels lower in the hierarchy. Crucially, this scheme implies a circular causality among inference, learning (or memory) and attention (or sensory attenuation). This means that inference, learning and precision depend upon each other in a way that is mandated by the reciprocal message passing of approximate Bayesian inference—of which predictive coding is a key, neuronally plausible, example (see Friston, 2008, more formal discussion).

The precision expected in any given context is mediated by modulating postsynaptic sensitivity, through top-down neuromodulatory mechanisms. For example, in the interoceptive domain, the sight of water could induce contextual cues that increase the precision of prediction errors signaling deviations from homoeostatic setpoints for blood osmolality. Thus, exteroceptive cues can bias attention to interoceptive thirst signals. Conversely, when hungry or thirsty, cues in the environment associated with satisfying these needs will be noticed, or take on a saliency that they would not otherwise demand. In a similar manner, prosocial (exteroceptive) cues such as eye gaze, a mother's face, tone of voice and biological movement may act as contextual modifiers to increase interoceptive signal precision and thereby promote emotional inferences early in development. Later, as the social brain emerges through developmental learning, these same cues will take on a salience that will capture attention. The neuromodulation underlying this vitally important control, first of interoceptive precision for accurate inference, then for the synaptic plasticity underlying learning of the generative model, and ultimately for appropriate context dependent top-down modulation that mediates attention and sensory attenuation we propose, depends upon oxytocin.

### 2.5 Oxytocin and precision

As discussed in previous papers on active inference, traditional neurotransmitters (dopamine, gamma-aminobutyric acid (GABA), glutamate, norepinephrine, and acetylcholine) can all modulate precision or the prediction error gain to promote the accurate inferences crucial to learning appropriate generative models (Friston, 2008, Friston et al., 2006, Friston et al., 2012, Stephan et al., 2009). The oxytocin system interacts with all of these neurotransmitter systems, allowing oxytocin to harness the neural mechanisms in place for enhancing attention (Levy et al., 1995), endowing signals with hedonic valence (Insel, 2003, Smeltzer et al., 2006), and augmenting associative learning and memory (Dluzen et al., 2000, Ferguson et al., 2000b, Ferguson et al., 2002, Ninan, 2011, Qi et al., 2012). Oxytocin modulates dopamine in the amygdala, nucleus accumbens, ventral tegmental area, and the substantia nigra (Love et al., 2012, Sanna et al., 2012), affects GABA release and subsequent neuronal activity in the hippocampus, hypothalamus and the central nucleus of the amygdala (Theodosis et al., 2006, Viviani and Stoop, 2008, Viviani et al., 2010, Zaninetti and Raggenbass, 2000). It also interacts with the acetylcholine and norepinephrine systems to both enhance social memory (Levy et al., 1995, Feifel et al., 2012, Ferguson et al., 2000b, Levy et al., 1995, Rimmele et al., 2009) and suppress aversive memories (Boccia and Baratti, 2000).

**Fig. 2:**
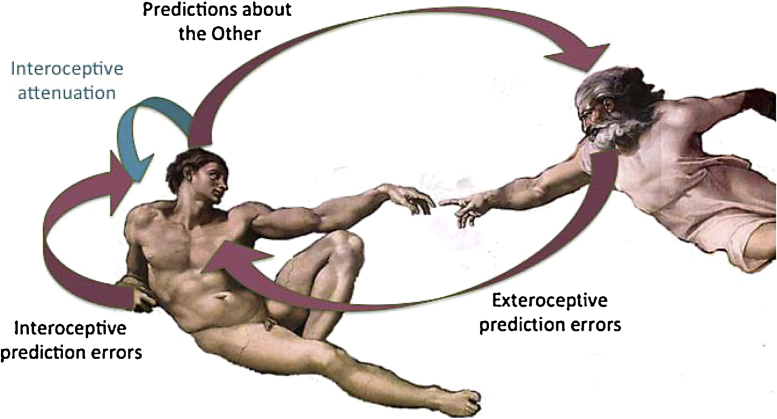
*Active inference and sensory attenuation.* This illustrates how sensory attenuation in active inference supports social interactions. Like action observation, social interactions require attenuation, not of proprioceptive information, but of interoceptive signals. In a predictive coding formulation of social engagement, interaction induced suppression of interoceptive signals, similar to motor induced suppression of proprioceptive signals, allows for the individual to infer the autonomic and emotional state of another, without eliciting the autonomic reflexes in self—in other words, the display of cognitive empathy without autonomic echopraxia or emotional contagion. The ability of interoceptive directed predictions to suppress otherwise inappropriate interoceptive prediction errors depends upon a complex generative model of the socio-emotional ‘self’. As explained in the text, the inferential learning necessary to create such a generative model and to deploy the appropriate predictions that emerge from the model, we argue, requires oxytocin-dependent neuronal plasticity during development.

In many treatments of predictive coding, *N*-methyl-d-aspartate (NMDA) type glutamate receptors are thought to mediate top-down neuromodulation (Friston, 2002). NMDA receptors also underlie some of the basic mechanisms of synaptic plasticity, including long-term potentiation and depression, for review see (Malenka and Nicoll, 1999). Oxytocin directly affects long term synaptic plasticity through its ability to initiate gene transcription and protein phosphorylation—as well as indirectly through its influence on NMDA receptors (Dubrovsky et al., 2002, Lin et al., 2012, Monks et al., 2003, Ninan, 2011, Tomizawa et al., 2003). Oxytocin may thus play a role similar to other modulators of NMDA receptor function, in mediating top down modulation—not only in the cortex, but also in the autonomic nervous system and associated subcortical regions of the brain. In what follows, we consider how the acquisition or learning of hierarchical models may depend upon oxytocin dependent selection of cues with interoceptive associations or significance in the environment. A failure to modulate the precision of interoceptive cues might therefore, as examined below, lead to the impaired acquisition of deep hierarchical models that underwrite a sense of self and the subsequent sensory attenuation crucial to developing central coherence and negotiating social interactions (Frith and Happe, 1994). See Fig. 2.

### 2.6 Oxytocin and learning

In predictive coding, learning represents the updating of associations and connection strengths responsible for generating top-down predictions. In other words, it underlies the acquisition or construction of a generative model that can explain away prediction errors more efficiently. In addition to learning associations among cues in the world, the generative model also has to learn contextual associations that selectively assign precision to appropriate processing channels. In terms of functional anatomy, the insular cortex holds a primary position in interoception and is thought to mediate the integration and associative learning that underlies higher level interoceptive inference (Craig, 2010, Damasio and Carvalho, 2013). The prefrontal cortex (e.g., anterior cingulate and ventromedial prefrontal region) can then integrate the ensuing representations as part of hierarchical inference that underlies emotional awareness (Seth, 2013).

Central to the hypothesis advanced in this review is the notion that oxytocin is necessary for contextualizing top-down predictions and the subsequent learning of multimodal generative models during development. Pertinent to learning, oxytocin receptor activation can operate on several time scales, facilitating both short-term neural activity and augmenting synaptic efficacy in a more enduring manner. Oxytocin modulates immediate neural transmission through G-protein activation and mobilization of internal calcium stores to enhance the release of traditional neurotransmitters (Raggenbass, 2001). On a longer time scale, oxytocin initiates a cascade of intracellular events with long-term consequences, through activation of protein kinases, thereby having lasting effects on synaptic efficacy (Tomizawa et al., 2003). Like NMDA receptors, multiple physiological events modulate the activity of oxytocin receptors. The presence of magnesium in the extracellular environment and proximate cholesterol in the membrane determine the affinity the receptor has for oxytocin (Gimpl and Fahrenholz, 2001). Oxytocin has positive, receptor mediated, feedback regulation of its own release (de Wied et al., 1993). Steroid hormones and methylation both alter expression levels during different stages of the lifespan (Sharma et al., 2012, Tribollet et al., 1989, Tribollet et al., 1990), and regulate tissue specificity of the oxytocin peptide and receptor (Amico et al., 1984, Amico et al., 1995, Amico et al., 2002, Crowley et al., 1993, Crowley et al., 1995, Gimpl et al., 2008). Collectively, these cellular mechanisms implicate oxytocin in integrating disparate information over multiple time scales; facilitating the plasticity implicit in the acquisition of complex generative models.

The etiology that emerges from a primary failure of interoceptive neuromodulation implicates both inference about states of the world and subsequent learning of the generative model supporting that inference. In this view, a failure of sensory attenuation is both cause and consequence of impoverished learning, because sensory attenuation itself has to be learned.

Oxytocin's role in social learning has been empirically demonstrated through genetic, pharmacologic, and behavioral studies. Oxytocin can potentiate acetylcholine dependent olfactory memory formation required for social recognition in rodents (for review see Bielsky and Young, 2004) and sheep (Levy et al., 1995), and has facilitatory effects on social memory in humans (Feifel et al., 2012, Hurlemann et al., 2010, Savaskan et al., 2008); yet, oxytocin can also suppress aversive memory formation in both humans (Petrovic et al., 2008), although see (Weigand et al., 2013), and rodents (Boccia and Baratti, 1999, Boccia and Baratti, 2000). In oxytocin peptide knockout mice, the absence of the neuropeptide oxytocin impairs olfactory-based social recognition, but leaves general spatial learning intact (Ferguson et al., 2000a). These mice not only do not form recognition memories for conspecifics, but also do not specifically recognize their mother's scent, yet their ability to remember the location of food through olfactory memory persists (Nelson and Panksepp, 1998). Furthermore, oxytocin appears to allow for the contextualization of social memories. Mice lacking the oxytocin peptide gained familiarity and could recognize social odors on a cotton ball, but could not contextualize this information, and thus were unable to recognize the conspecific when presented with the owner of the odor (Winslow and Insel, 2002).

### 2.7 Oxytocin and emotional affordance

In summary, based on oxytocin mediated associative learning during attachment early in development and the persistent physiological role of oxytocin on top-down modulation and sensory attenuation, the oxytocin system is well placed to actuate the salience of socially relevant cues in the environment. Clinical evidence from studies administering exogenous oxytocin speaks to this role. Several studies have demonstrated that oxytocin administered to healthy individuals facilitates facial emotional recognition (Domes et al., 2007b, Rimmele et al., 2009, Savaskan et al., 2008), increases attention to the eye region of the face (Guastella et al., 2007), enhances the rewarding valence of social stimuli (Hurlemann et al., 2010), increases trust (Baumgartner et al., 2008, Keri and Kiss, 2011, Kosfeld et al., 2005), enhances empathy (Bartz et al., 2010), encourages generosity (Zak et al., 2007), facilitates cooperation (Rilling et al., 2012), and promotes a willingness to interact with others (Andari et al., 2010). Collectively, these results suggest that oxytocin enhances the saliency of socially relevant stimuli; indeed, imaging studies have revealed the functional anatomy of its effects on saliency (Andari et al., 2014, Domes et al., 2007a, Jack et al., 2012, Wittfoth-Schardt et al., 2012).

**Fig. 3:**
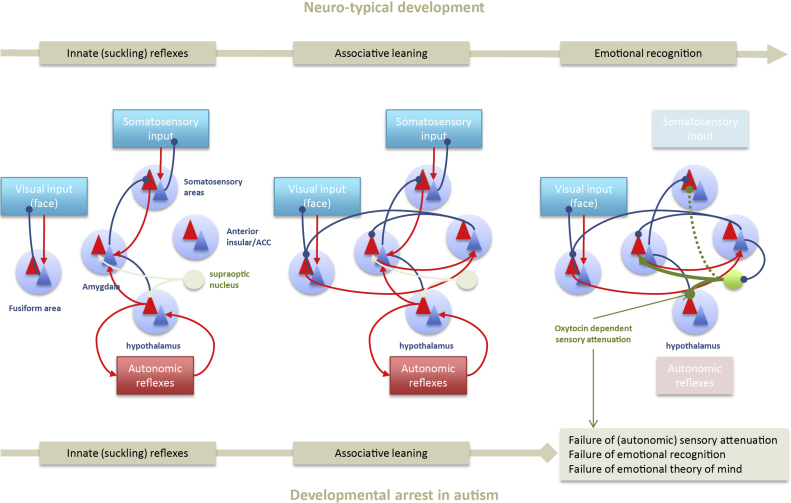
*Oxytocin and the development of emotional affordance.* This schematic describes normal (and autistic) neurodevelopmental trajectories, in terms of (simplified) neural architectures underlying predictive coding of autonomic (emotional) signals. The three panels illustrate the development of associative connections we imagine underlie the acquisition of emotional responses during three stages of development. The anatomical designations should not be taken too seriously—they are just used to illustrate how predictive coding can be mapped onto neuronal systems. In all of these schematics, red triangles correspond to neuronal populations (superficial pyramidal cells) encoding prediction error, while blue triangles represent populations (deep pyramidal cells) encoding expectations. These populations provide descending predictions to prediction error populations in lower hierarchical levels (blue lines). The prediction error populations then reciprocate ascending prediction errors to adjust the expectations (red lines). Arrows denote excitatory connections, while circles denote inhibitory effects (mediated by inhibitory interneurons). **Left panel**: in the first panel, connections are in place to mediate innate (epigenetically specified) reflexes – such as the suckling reflex – that elicit autonomic (e.g., vasovagal) reflexes in response to appropriate somatosensory input. These reflexes depend upon high-level representations predicting both the somatosensory input and interoceptive consequences. The representations are activated by somatosensory prediction errors and send interoceptive predictions to the hypothalamic area—to elicit interoceptive prediction errors that are resolved in the periphery by autonomic reflexes. Oxytocin is shown to project to the high-level representations (the amygdala) and the hypothalamic area, to modulate the gain or precision of prediction error units. In this schematic, its effects are twofold: oxytocin attenuates the gain of hypothalamic prediction error units and augments the gain of higher level units. **Middle panel**: this shows the architecture after associative learning, during which high-level representations in the anterior cingulate or insular cortex have learned the coactivation of amygdala representations and exteroceptive cues (e.g., the mother's face during suckling). These high-level representations now predict the exteroceptive visual input and (through the amygdala) somatosensory and autonomic consequences. **Right panel**: in this schematic, visual input (e.g., the mother's face) is recognized using the high-level representation in the anterior insular or cingulate cortex. However, in this case, interoceptive prediction error is attenuated so that it does not elicit an autonomic response. In other words, although the high-level emotional representation is used to recognize exteroceptive cues, lower-level transcortical reflexes are inhibited. In autism, we presume that oxytocin is deficient, such that sensory attenuation is impaired – leading to disinhibition of autonomic responses and the failure to recognize a mother's face in any other context – other than during suckling. This failure of sensory attenuation may underlie autonomic hypersensitivity, failure of emotional recognition, attention to emotional cues, theory of mind and central coherence. The dotted green line in this figure acknowledges that there may not be any direct projections from the origin of oxytocin cells (in the supraoptic and paraventricular nuclei of the hypothalamus) to secondary or primary somatosensory cortex. (For interpretation of the references to color in this figure legend, the reader is referred to the web version of this article.)

To learn emotional affordance during infancy, neuromodulatory mechanisms must be in place to establish the associations between interoceptive cues, such as homoeothermy, or satiety, and exteroceptive cues, such as a primary care giver's face and touch. In the typically developing infant, oxytocin may be a key modulator that facilitates this associative learning—through selectively augmenting and attenuating interoceptive prediction errors. For instance, associative learning might start by establishing a connection between the suckling reflex and the smell of the breast. Oxytocin is known to promote the acquisition of olfactory based social recognition memory (Ferguson et al., 2002) and the suckling reflex (Wrobel et al., 2010). Through predictive coding, this association would then become assimilated into more complex models – with greater hierarchical depth – that would eventually lead to emotional recognition of the primary care giver's face and infant attachment. See Fig. 3.

Attachment contributes to the survival of the species and the resulting selection pressure – that sustains a strong attachment style – may have shaped the neurobiology that drives social interactions (Bartels and Zeki, 2004). As the mediator of lactation, oxytocin has an obvious effect on the maternal infant bond by facilitating the primitive role of feeding; in both the mother, through lactation, and in the newborn through the suckling reflex (Febo et al., 2005). Besides feeding, eye contact is one of the first initiators of a shared attachment that lays the foundation for subsequent, mutually reinforcing, relationships later in development. The emotional affordance assigned to eye gaze in neurotypical infants most likely results from early associations arising from eye contact between mother, or primary care giver, and the infant. Eye contact with the primary care giver often elicits the first evidence of social smiling in infants and increases satisfaction in the care giver (Robson, 1967). Typical infants preferentially respond to eye gaze by 3–4 months and can be soothed solely through eye contact. Oxytocin may play a role in the normal development of this aspect of attachment, as several studies now demonstrate that acute intranasal administration of oxytocin improves eye contact both in normal controls (Guastella et al., 2007, Tollenaar et al., 2013), although see (Lischke et al., 2012a) and in autism (Andari et al., 2010, Domes et al., 2013, Guastella et al., 2010). By optimizing the precision of interoceptive cues relating to satiety, satisfaction, and relief – that are preceded by eye contact with the primary care giver – oxytocin could play a fundamental role in establishing the connections necessary for recognizing that interoceptive states are caused by these interactions. This learning corresponds to acquisition of generative models that imbue eye contact (and other affiliative cues) with emotional affordance or salience throughout life.

## 3 Active inference

### 3.1 Active inference and homeostasis

**Fig. 4:**
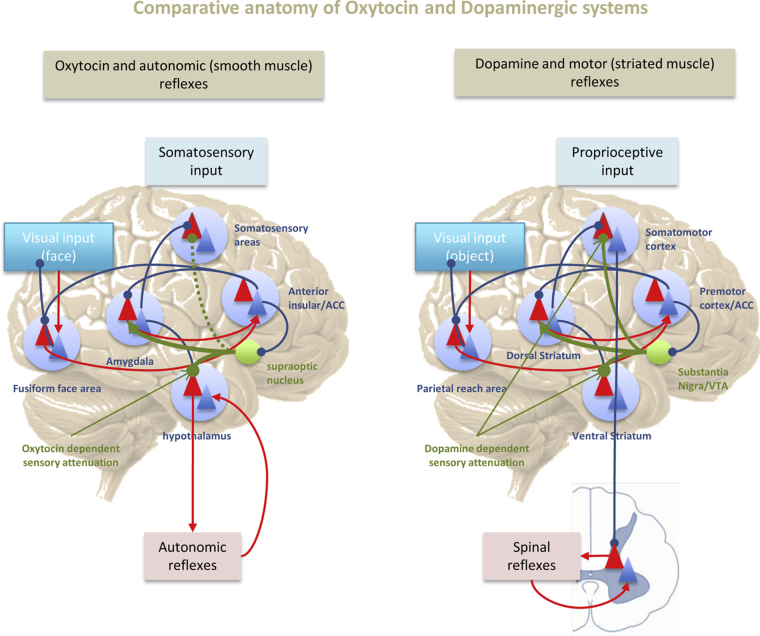
*Oxytocin dependent neuromodulation compared to dopamine.* This schematic uses the same format as Fig. 3 to illustrate the similarities between the role of oxytocin in modulating the precision of interoceptive signals and the role of dopamine in modulating proprioceptive signals. Again, the anatomy and details should not be taken too seriously; however, the overall architecture depicted here emphasizes their similarity – in terms of opposing neuromodulatory roles that could increase the precision of higher-level prediction errors, while decreasing the precision of lower-level prediction errors. For oxytocin, we have illustrated this by an augmentation of gain in the amygdala and an attenuation of sensory precision in the hypothalamic region. For dopamine, the same complementary effects are illustrated in the dorsal and ventral striatum, mediated by D1 (go pathway) and D2 (no-go pathway) receptors, respectively. In both cases, context sensitive neuromodulatory signaling is listed by descending projections (predictions) from cortical regions involved in emotional regulation (oxytocin) or action selection (dopamine). Deficits of oxytocin – we propose – lead to a failure of interoceptive processing, while deficits of dopamine (for example in Parkinson's disease) compromise proprioceptive processing and the initiation of action.

Active inference refers to the fulfillment of predictions through action (Friston et al., 2010), (see middle panel of Fig. 1). In the setting of interoceptive inference, it calls on models of the internal or emotional self to predict the action necessary for the fulfillment of interoceptive drives. Interoceptive action can either take the form of a behavior to satisfy a drive or the deployment of an autonomic response to re-establish homeostasis (see right panel of Fig. 1). In this setting, expectations about our emotional and embodied self generate descending predictions that engage autonomic reflexes and provoke behaviors to orchestrate an integrated response to biological drives and homeostatic requirements. In interoceptive active inference, the fronto-insular cortex – with the anterior cingulate cortex – acts much like a ventral extension of the motor cortex for the autonomic nervous system (Kaada et al., 1949, Nimchinsky et al., 1999, Seth, 2013). This brain region possess large fast-acting von Economo neurons (Allman et al., 2011, Nimchinsky et al., 1999, Seeley et al., 2012, von Economo, 1926, von Economo and Koskinas, 1929) that, like Betz cells in the motor cortex, reside in cortical layer 5 of the fronto-insular cortex and send large descending projections to lower structures, including the hypothalamus and brainstem nuclei. These sorts of projections conform to the requirements established by predictive coding formulations for the transmission of descending predictions (Adams et al., 2013) and allow the anterior insula together with the anterior cingulate to control autonomic responses. This control corresponds to specifying equilibrium set-points with top-down interoceptive predictions (Seth, 2013), much like active inference in the motor system (Friston, 2010, Friston et al., 2012). The idea here is that descending predictions of interoceptive sensations provide the set point for autonomic reflexes, while top-down (corticospinal) predictions of proprioceptive sensations provide the equilibrium points for classical motor reflexes—and thereby control motor trajectories. Fig. 4 highlights the similarity between the neuromodulatory role of dopamine in proprioception and oxytocin in interoception. These neuromodulators play a crucial role in gain control and sensory attenuation that may be fundamental for homoeostasis and behavioral allostasis, respectively.

Applying active inference in the interoceptive domain enables the autonomic nervous system to regulate homeostasis by minimizing interoceptive prediction errors – very much like the homoeostatic formulation of Ashby (1947), and the free energy principle (Friston et al., 2010). Oxytocin, through its effect on the hypothalamus and the NTS in the brain stem can nuance autonomic reflexes (Coote, 2005, Michelini, 2007). Furthermore, oxytocin is known to modulate heart rate variability (Kemp et al., 2012), respiration (Kc and Dick, 2010), and the sexual response (Argiolas and Melis, 2005, Melis and Argiolas, 2011, Witt and Insel, 1992), for review see (Witt, 1997), suggesting a key role in autonomic homeostatic control.

### 3.2 Active inference and behavior

Many of the neuropeptides that participate in the autonomic control of homeostasis exert complementary effects in the central nervous system by initiating and regulating allostatic behaviors. Oxytocin, as one of many neuropeptides involved in homeostasis, does not operate alone in modulating behavior; but, oxytocin may play a privileged role in the autonomic control and action selection related to reproduction and hence the social, affiliative, and emotional behaviors that subserve physiological and emotional equilibrium. Through its modulation of central pattern generators associated with movement, oxytocin can elicit instinctual behaviors such as yawning (Sanna et al., 2012), tongue action (Wrobel et al., 2010) copulatory movements (Garrison et al., 2012, Wagenaar et al., 2010), social vocalizations (Bass and Remage-Healey, 2008), and locomotion (Barrière et al., 2005). These findings support a modulatory role for oxytocin in the reflexive action selection necessary to fulfill top-down predictions relevant for instinctual affiliative behaviors, and ultimately, social interactions. So why would neuromodulation be so important for maintaining homoeostasis and fulfilling instinctual drives? We address this next in terms of *sensory attenuation*.

## 4 The ‘self’, sensory attenuation and active inference

### 4.1 Oxytocin and generative models of the self

Concepts of the ‘self’ have a long history in western philosophy dating back to Plato's *Republic* (Plato, 360 B.C.E.), and re-emerging as a central concept in modern psychology through the writings of James (1890). In the context of this review, a multifaceted model of the ‘self’ encompasses individuation (self-other discriminability, ego boundaries), bodily self-awareness, emotional self-awareness, continuity (across time) and free will (agency). At the level of cognitive psychology, the possible role oxytocin plays in acquiring generative models of the self has been less examined. However, oxytocin has notable effects on early attachment with a primary caregiver, and given the role attachment plays in forming early concepts of the ‘self’ (Bowlby, 1977), oxytocin may participate in the construct of such a model. Much has been written about the mother in maternal-infant attachment, but less scrutiny has focused on the infant as an active participant in this interaction. Oxytocin has well documented influence on both maternal physiology and behavior, for review see (Carter, 2014), and even enhances paternal care giving (Weisman et al., 2014). Oxytocin in the infant, however, also underwrites the development of this bond. Genetically altered mice – that do not possess the oxytocin gene and are therefore deficient in endogenous oxytocin – do not vocalize like normal pups when separated from their mother, suggesting that oxytocin helps mediate the processing fundamental to inferring, or recognizing, the state of separation or the ability to communicate distress (Winslow et al., 2000). Either explanation indicates that oxytocin mediates key aspects of behavior in infancy that subserves attachment. Additional results implicating oxytocin in the cognitive constructs of the self include the finding that administration of oxytocin in humans can enhance the awareness of demarcations between the self and others (Colonnello et al., 2013), and that oxytocin differentially modulates reactions to “in-group” versus “out-of-group” individuals, in measures of empathy, trust, and cooperation (De Dreu, 2010, De Dreu et al., 2011, De Dreu et al., 2012). The differentiation between sameness and otherness suggests oxytocin enhances a sensitivity to individuation.

### 4.2 Oxytocin in perinatal development

During the perinatal period, oxytocin triggers an important change in the physiology of GABAergic neurons. Maternal oxytocin rises just before parturition, crosses the placenta, and mediates a change in the resting neuronal chloride concentration in the central nervous system of the fetus by inactivating the sodium–potassium–chloride-transporter (NKCC1) (Tyzio et al., 2006). Prior to parturition, GABA neurons have a relatively high resting chloride reversal potential, due to the activity of NKCC1 transporting chloride ions into the cell. Given the resulting high internal chloride concentration, activation of GABA-A receptor allows negatively charged chloride ions to flow out of the cell, depolarizing the membrane and causing GABA to function as an excitatory neurotransmitter. GABA neurons dominate in the developing brain and affect many aspects of brain maturation—guiding neuronal migration, stimulating the growth and formation of early synapses (Wang and Kriegstein, 2009) and initiating the majority of the calcium-dependent oscillations in the hippocampus and in other areas of self-generating neuronal activity (Bonifazi et al., 2009, Fiumelli and Woodin, 2007). In the mature brain, however, GABA-A receptors have inhibitory effects on neurons by allowing chloride to flow passively into the cell—following a high concentration gradient of external chloride relative to intracellular levels. In the mature brain, this gradient is produced by the activity of the opposing chloride potassium co-transporter, KCC2, which extrudes chloride out of the cell in an electro-neutral fashion.

During the embryonic and prenatal stages of development, the NKCC1 transporter dominates, and more chloride ions are transferred into the cell than out of the cell. The KCC2 transporter does not become active until later in development, thus during embryonic stages, the sole activity of the NKCC1 creates a chloride gradient that allows GABA transmission to excite cells instead of inhibit them. Oxytocin dramatically changes the activity of GABA neurons with the onset of labor by inactivating the NKCC1 transporter. In rats, during parturition – and for a few postnatal days – GABA neurons become inhibitory. As oxytocin levels remit, GABA transmission transiently becomes excitatory again, until post-natal expression of the KKC2 transporter begins to exert its opposing effects on the chloride gradient, once more allowing GABA to assume it mature role in the brain as an inhibitory neurotransmitter (Ben-Ari et al., 2012). The peripartum window of GABA mediated inhibition in the CNS protects the newborn brain from labor and delivery induced transient hypoxia and produces mild analgesia (Ceanga et al., 2010).

This remarkable effect of oxytocin on GABAergic transmission has not been studied in the human brain, but several physiologic findings suggest that a similar mechanism takes place in the human newborn. For example, infants exposed to higher levels of oxytocin through vaginal deliveries resist pain and can withstand hypoxia more effectively (Mazzuca et al., 2011). In addition, perinatal seizures, which are notoriously difficult to treat, respond poorly to the usual drugs that promote GABAergic transmission (Connell et al., 1989), yet administering bumetanide, a NAKCC1 inhibitor, can quell seizure activity when other antiepileptic drugs have failed (Dzhala et al., 2008, Pressler and Mangum, 2013). The success of this medication in perinatal seizures suggests that GABA channels in the human newborn are also capable of acting in both an excitatory and inhibitory manner, depending on the activity of the chloride transporters and the resultant chloride reversal potential. This intervention, in a small trial, has also shown moderate success in ameliorating some clinical symptoms of autism (Lemonnier et al., 2012).

### 4.3 Interoceptive attenuation and homeostasis

A potential paradox exists when applying predictive coding to the reflexive suppression of proprioceptive or interoceptive prediction errors. This paradox results from the fact that the simplest way to resolve sensory prediction errors would be to change top-down predictions. However, this would preclude any remedial movement or autonomic response. It is therefore necessary to attenuate the precision of ascending sensory prediction errors to enable descending predictions to be fulfilled by peripheral reflexes. This permissive suspension of sensory precision takes the form of sensory attenuation (Brown et al., 2011). Sensory attenuation refers to the reduced discriminability of sensations caused by self-made acts (for review, see Sommer and Wurtz, 2008). From the perspective of active inference, the (psychophysical) phenomenon of sensory attenuation is mediated by the (physiological) attenuation of sensory precision or gain. Successful self-generated actions or behaviors require sensory attenuation. Examples of such sensory attenuation include the suppression of self-generated visual sensations during saccadic eye movements (Hohwy et al., 2008, Miles and Evarts, 1979, Wurtz et al., 2011), or the attenuated perception to the sound of our own voice (Aliu et al., 2009, Paus et al., 1996). In the interoceptive domain, sensory attenuation accounts for why self-generated movements seldom tickle or produce sensual pleasure; whereas, a similar touch by someone else can evoke more hedonic sensations. The anterior insula, particularly the right anterior insula, is implicated in a sense of bodily ownership and the recognition of self generated movement and touch (Karnath and Baier, 2010)—and thus may participate in the sensory attenuation necessary for active inference in the interoceptive domain. See Fig. 2.

### 4.4 Interoceptive attenuation and homeostasis

We have seen that the attenuation of interoceptive precision may be necessary for self-generated action or behavior that relates to interoception and the maintenance of homeostasis, an interoceptive form of action. The role of precision and attenuation of interoceptive prediction errors may therefore be fundamental in the organization and selection of autonomic reflexes. In other words, homoeostatic regulation may require the temporary suspension of interoceptive precision—attending away from the current interoceptive state of the body, such that top-down predictions can elicit peripheral sympathetic and parasympathetic reflexes. That homeostatic regulation depends upon a sense of bodily ownership supports the notion that homeostatic action is an integral part of active (embodied) inference.

The finding that the rubber hand illusion can affect autonomic thermoregulatory control over the limb that has been transiently forsaken, suggests that a sense of bodily ownership, or a well-informed top down prediction, facilitates interoceptive control (Moseley et al., 2008), yet see (Paton et al., 2012, Rohde et al., 2013). The right insula has been implicated in this sense of ownership and autonomic control, together with the anterior cingulate and the fronto-insular cortex. Interestingly, von Economo neurons – which reside in these areas and locate more abundantly in the right hemisphere – may expedite transmission necessary for fast autonomic regulation through interoceptive predictions (Butti et al., 2013) and, possibly, sensory attenuation. The key role of sensory attenuation in permitting the expression of voluntary (striatal muscle) and visceral (smooth-muscle) reflexes explains why neuromodulation plays a crucial role in active inference—and hints at the pathologies that would follow from a neuromodulatory failure of sensory attenuation (cf., the role of Dopamine in Parkinson's disease, Fig. 4).

Evidence for oxytocin's ability to attenuate interoceptive precision in the context of self-generated action – and thereby facilitate autonomic responses – exists in both the animal and clinical literature. In rodents, oxytocin promotes GABA mediated inhibitory activity in the central nucleus of the amygdala, the main projection from the amygdala to brain stem nuclei and the hypothalamus in the fear circuit (Viviani et al., 2010) in preparation for action. In humans, acute administration of intranasal oxytocin can increase parasympathetic tone, reduce arousal (Grewen and Light, 2011), and ameliorate the stress responses (Gamer and Buchel, 2012, Grewen and Light, 2011, Kemp et al., 2012, Pitman et al., 1993). It can have general anxiolytic properties in socially stress inducing situations, (Alvares et al., 2010, Alvares et al., 2012, de Oliveira et al., 2012a, de Oliveira et al., 2012b, Heinrichs, 2001) and can augment the beneficial effects of other stress reduction interventions (Guastella et al., 2009, Heinrichs et al., 2003). Functionally, intranasal oxytocin decreases both amygdala activation as well as coupling between the amygdala and the brainstem (Domes et al., 2007a, Kirsch et al., 2005, Wittfoth-Schardt et al., 2012); although – consistent with its facilitatory role in maternal behavior – oxytocin can also increase amygdala reactivity to threatening stimuli in females to activate protective behavior (Lischke et al., 2012b). These two seemingly contradictory effects of oxytocin make sense if viewed through the lens of predictive coding. As the mediator of the necessary sensory attenuation required for implementing self-generated action in the social emotional domain, oxytocin prepares for action, whether by reducing fear to encourage approach, or maintaining arousal to facilitate protection of offspring.

### 4.5 Interoceptive attenuation in social exchanges

Sensory attenuation may be fundamental to social interactions as well as homeostatic regulation. Oxytocin could exert multiple influences on prosocial and affiliative action or behavior through sensory attenuation. An ability to act, whether through movement or behavior, often requires sensory attenuation. In this context, interoceptive sensory attenuation pertains to shifting attention outward, away from the self. By reducing anxiety and self-absorption, oxytocin dependent sensory attenuation would promote approach behaviors (Petrovic et al., 2008, Wittfoth-Schardt et al., 2012), although see (Scheele et al., 2012). Additionally, by diminishing the hedonic pleasure of self-induced stimulation, interoceptive sensory attenuation would motivate the behaviors necessary to seek out interactions with others. Mice lacking the oxytocin receptor spend more time self-grooming and less time participating in reciprocal grooming (allo-grooming) than wild type mice (Pobbe et al., 2012), suggesting self-stimulation may be more rewarding without the sensory attenuation that a functioning oxytocin system might provide. Rhesus monkeys raised without a primary care-giver have lower oxytocin cerebral spinal levels which correlate with reduced allo-grooming, more isolation and increased repetitive stereotypies (Winslow et al., 2003).

In addition to initiating social interactions, oxytocin may enable social success once engaged, through interoceptive sensory attenuation. As with action observation, social interactions require the balanced attention to exteroceptive cues and attenuation of internal cues, in order to make inferences about others. Besides an attentional focus on salient biologic details, inference about the visceral or emotional state of the ‘other’ requires a careful sensory attenuation of our own interoceptive signals—in order to infer the emotional or autonomic state of others without interference from our own interoceptive cues. A successful social interaction therefore requires both the attribution of salience to exteroceptive cues, such as eye gaze and tone of voice in the ‘other’ and the ability to suppress inappropriate interoceptive cues. These abilities have to be learned as part of the generative model: the neuromodulation that attributes precision to prosocial cues must rely on prior expectations about facial expressions, tone of voice, and bodily stance that have been accrued through experience, and successful sensory attenuation of our own interoceptive signals depends on learning fine grained generative models of the self-learning that we suggest depends on oxytocin-dependent modulation of interoceptive cues.

### 4.6 Sensory attenuation, empathy and oxytocin

Interoceptive sensory attenuation may also scaffold the processing imperative for the emergence of cognitive empathy and compassionate behavior. In the hierarchy of emotional reactions to another, emotional contagion represents the most primitive form of empathy and entails experiencing their emotional state. The transformation of affective contagion into cognitive empathy (Theory of Mind) requires successful attenuation of one's own emotional (autonomic) reaction and, implicitly, maintaining the boundaries of the self. Thus, cognitive empathy that does not automatically elicit an autonomic echopraxia or emotional contagion requires a well-developed sense of self (Decety and Lamm, 2006). Exogenous oxytocin improves empathy and the ability to infer the mindset of others (Bartz et al., 2010, Domes et al., 2007b, Hurlemann et al., 2010). Trials of oxytocin in autism have produced both acute (Andari et al., 2010, Guastella et al., 2010) and long term (6 weeks) (Anagnostou et al., 2012) improvements in performance on inferring the emotional state of others in the “Reading the mind through the eyes test”. A genetic study also supports the association between autonomic attenuation and empathy—by demonstrating that oxytocin receptor variants contribute to behavioral measures of empathy and stress reactivity in a synchronous manner (Rodrigues et al., 2009).

In rodents, oxytocin selectively facilitates GABA transmission in a subpopulation of amygdala cells, decreasing the freezing response, while maintaining the visceral appreciation of fear (Viviani et al., 2011). One can imagine that freezing might be self-protective, but that maintaining fear, while having the capacity to act, might allow one to help others. It is tempting to speculate that this represents the transformation from emotional (contagion) to cognitive empathy—suggesting that oxytocin, via interoceptive sensory attenuation, facilitates the progression from affective experience to compassionate behavior, even in a rat.

### 4.7 Context dependent modulation

In the proposed model, differences in context can explain some of the apparently contradictory effects of oxytocin. Although over-simplified, the dichotomy between perception and action dictates whether oxytocin behaves to either attenuate or enhance interoceptive signals. Oxytocin participates in interoceptive sensory attenuation in the context of self-generated action with interoceptive consequences, which, in our minds, refers to homeostatic regulation, allostatic behaviors to fulfill drives, and social interactions. To accomplish these goals, oxytocin dependent sensory attenuation generally increases the precision of higher level predictions, based on learned generative models of the self, to dampen unhelpful interoceptive prediction error signals that would otherwise preclude action by causing a revision of the prediction, or by promoting echopraxia. Thus, in the context of self-generated action, one would expect oxytocin to dampen interoceptive signals. The evidence from Viviani's experiment, described above – where oxytocin mediated a switch from a freezing response to promoting action in rats – supports this prediction (Viviani et al., 2011). In contrast, in the absence of action, the model would predict that oxytocin would augment interoceptive signal prediction errors through top-down modulation; much like attention can enhance visual saliency. A recent study provides empirical support for the notion that oxytocin enhances interoceptive signals in such a context dependent manner. In human males, intra-nasal oxytocin administration increases the hedonic pleasure associated with light touch in a context dependent manner. Despite identical touch stimulation, oxytocin increased the pleasurable experience of touch when male heterosexual subjects assumed an attractive female performed the action as compared to a male. A concomitant increase in insular and pregenual anterior cingulate cortex activation mirrored the difference in perception, suggesting oxytocin exerts its effects in this context through top-down modulation, and this top-down modulation correlates with activation in the anterior insula and pregenual ACC (Scheele et al., 2014).

### 4.8 Failures of interoceptive attenuation

In summary, without adequate attenuation of interoceptive precision, internal prediction errors cannot be used to inform multimodal representations of emotional states that distinguish self from non-self, nor would the normal associations between emotional states and exteroceptive cues be learned. This would result in confusing the interoceptive state of self from others (emotional contagion), diminished emotional affordance of exteroceptive cues (failing to assign salience to a mother's face), and poorly formed representations of the ‘self’ (poor ego boundaries). This means that a dysfunctional oxytocin system could prevent the sensory attenuation necessary to develop a mindfulness of others, to engage in social interactions and to learn complex behavioral repertoire is based on social imitation. We therefore suggest that autism is a result of the following etiological sequence:

• A primary disruption of the oxytocin system interferes with accurate interoceptive inference early in development.
• A developmental neuromodulatory deficit in oxytocin impairs the modulation of interoceptive signals and prevents their contextualization. This contextualization is necessary to learn associations between interoceptive cues and their exteroceptive, kinesthetic and proprioceptive precedents.
• A failure to learn context-sensitive associations between interoceptive and exteroceptive cues (in particular, cues from the primary care giver such as eye gaze, vocal prosody, and biological movement), precludes context sensitive attributions of salience to socially and emotionally relevant stimuli.
• Impaired integration of interoceptive signals with salient information from other sensory domains prevents the infant from acquiring a higher level representation of ‘self,’ embellished with an emotional dimension that is distinct from ‘other’.
• An impoverished sense of ‘self’ compromises interoceptive sensory attenuation. Impaired interoceptive sensory attenuation undermines self-generated action and behavior in the interoceptive domain, including: homeostatic regulation, self-stimulation, coordinated biological movements, and imitation based behaviors, eventually leading to difficulties with theory of mind and cognitive empathy.

In the final section, we turn to the evidence for the deficits predicted by these theoretical considerations. We first consider the evidence for the primary pathophysiology, which we suppose is a failure to prescribe appropriate precision to interoceptive signals due to abnormal oxytocin-dependent neuromodulation and the ensuing diminished emotional affordance of social and emotional cues in the environment and impoverished generative models of the self. We then look at the evidence for the secondary consequences of this failure; namely, poor interoceptive sensory attenuation and the resulting impairments associated with this dysfunction.

## 5 Evidence for interoceptive deficits in autism

### 5.1 Failure of interoceptive inference: Alexithymia in autism

Given the etiological sequence described above, a (primary) failure to modulate interoceptive precision would disrupt the context-sensitive assimilation of prediction error signals necessary to acquire generative models for inference about the emotional state of self and others. Impaired interoceptive inference would therefore give rise to alexithymia, or an inability to recognize and define one's own emotions. Several studies confirm that individuals with autism have difficulty understanding their interior landscape and cannot identify or name their own emotions with appropriate nuances, independent of their language capabilities (Bernhardt et al., 2013, Hill et al., 2004, Samson et al., 2012), often to a degree that reaches clinical alexithymia, for review see (Bird and Cook, 2013). Although no study has yet assessed oxytocin's ability to improve alexithymia in autism, single doses of oxytocin can enhance emotional understanding, as measured by the Reading the Mind through the Eyes test (RMET), in males diagnosed with autism (Andari et al., 2010, Guastella et al., 2010). As discussed below (in Section 5.9), individuals with autism do not lack affective empathy and can experience the emotions of others through emotional contagion. The improved scores on the RMET with oxytocin in autism could therefore reflect the selective attenuation of interoceptive signals enabling them to call on high-level emotional constructs without engaging autonomic reflexes. Consistent with this interpretation is the observation that oxytocin administration improves social cognition, or emotional inference, to a greater degree in those individuals who were more impaired in their ability to express their own emotions at baseline (Luminet et al., 2011).

### 5.2 Emotional affordance deficits in autism

Deficits in emotional affordance have been documented in autism since Kanner (1943) early description—when he reported that the autistic child, “never looks at anyone in the face”. Diminished attention to emotional stimuli contributes to clinical impairment, may interact with the development of other symptoms, involves both the visual and auditory realm and, by many accounts, appears within the first post-natal year (Ozonoff et al., 2010). A debate on whether poor attention to socially salient stimuli represents disinterest or active avoidance emerged with the recognition of the disease (Hutt and Ounsted, 1966, Richer and Coss, 1976). A dysfunctional oxytocin system could account for both sides of this debate through oxytocin's ability to impart salience to social cues and to nuance autonomic regulation. The need to avoid social interactions in autism could result from a failure to attenuate interoceptive precision when confronted with a confusing onslaught of overwhelming stimuli and will be discussed in the sections below on autonomic regulation (Section 5.6) and empathy (Section 5.9). Conversely, the apparent disinterest in socially relevant stimuli could reflect a different manifestation of the same inability to attenuate interoceptive signals. The resulting failure to contextualize interoceptive signals with exteroceptive cues that contain important social information, will impair the child's ability to prescribe precision (attention) to appropriate social cues. Conflicting evidence has emerged regarding attentional dysfunction in autism, which pervades several dimensions of cognition, (for review see Keehn et al., 2013); yet, many of the difficulties appear to center on a common deficit of diminished expected precision for socially relevant stimuli—a symptom consistent with a failure of precision-dependent attentional selection in both the visual and auditory domains. If these deficits derive from a primary failure to contextualize interoceptive signals and associate these signals with exteroceptive cues, as advanced by our model, the processing difficulties in selectively attending to social stimuli should rest on a failure to generate top down predictions that would typically augment signals from the channels processing these cues in the environment.

### 5.3 Visual affordance deficits

In the visual domain, autistic individuals maintain less eye contact over the course of their development; first with their mothers or primary care givers, and then with their peers (Hutt and Ounsted, 1966, Richer and Coss, 1976), for review see (Senju and Johnson, 2009) and do not selectively attend to social cues in the environment. Several ERP studies distinguish autism from typical development by a lack of preference for, or diminished attention to, social cues and weak orienting responses to faces and eye contact (Dawson et al., 1988, Dawson et al., 2002, Dawson et al., 2004, Gunji et al., 2009, Webb et al., 2006, Webb et al., 2012). FMRI studies also suggest that autistic individuals process faces, facial expressions, and eye contact differently in relation to age matched and IQ equivalent controls (Agam et al., 2010, Dalton et al., 2005, Davies et al., 2011, Greene et al., 2011, Nummenmaa et al., 2012, Pitskel et al., 2011, Zürcher et al., 2013). These differences appear to reflect a diminished engagement of face selective regions (Critchley et al., 2000, Davies et al., 2011, Kleinhans et al., 2010, Pelphrey et al., 2007). In predictive coding, this represents a selective failure in top-down predictions in visual streams functionally specialized for emotional and social cues. The findings from functional imaging, together with behavioral studies designed to test the integrity of top-down versus bottom-up processing, substantiates the notion that abnormalities of social perception in autism result from top-down deficits (Akechi et al., 2009, Gunji et al., 2009, Loth et al., 2010, Neumann et al., 2006, Webb et al., 2006, Webb et al., 2012).

### 5.4 Auditory affordance deficits in autism

Besides eye contact and facial emotional processing, human interactions rely heavily on communication through the auditory domain as well. Vocal communication includes not just language, but the prosody embedded in speech (Ross and Monnot, 2008). The pervasive language impairment observed in autism is consistent with deficits in auditory processing of social and biological stimuli (Dawson et al., 1998, Klin, 1991, Klin, 1992); for review, see (Jeste and Nelson, 2009). Studies of auditory processing in autism suggest that speech sounds do not engage the attention required for developing an early acumen for language (Ceponiene et al., 2003, Whitehouse and Bishop, 2008). This lack of attention to speech, preference for non-speech sounds (Kuhl et al., 2005) and impairments with speech discrimination (Lepisto et al., 2005) may, over time, have the self-reinforcing consequence of reducing the acquisition of generative models necessary for speech and language.

These findings, in both the visual and auditory domains, suggest that autistic individuals do not acquire the predictive and attentional predilection for biological and social stimuli. This is consistent with a selective failure to learn associations – in hierarchical models – that jointly predict exteroceptive and interoceptive consequences of social encounters. Endogenously controlled shifts in attention enhance the perception of external stimuli and increase the efficiency of sensory processing (Driver and Frith, 2000). One would predict therefore, that the observational learning from a stimulus that engendered more attention would be increased; whereas, a selective paucity of attention to eye gaze, faces, phonemic sounds and other biological stimuli could cause impoverished social learning.

The literature confirms that attentional deficits result in learning delays and disabilities (Elsabbagh et al., 2014). Observational learning in infants underlies the explosive acquisition of behaviors that develop within the first three years of life, including language, social communication, and fluid motor sequences. In summary, if autistic infants have a selective problem in predicting and attending to social and biologic stimuli in the environment, then there could be serious consequences that impede their learning of social contingencies and skills that could account for many of the symptoms that characterize the disorder. We propose that the failure to predict emotional and social salience in the environment results from a diminished ability both to learn associations between exteroceptive and interoceptive cues and to successfully attenuate autonomic reactivity in a context-sensitive fashion.

### 5.5 Evidence for an underdeveloped sense of self in autism

An inability to incorporate specific interoceptive signals into complex generative models of the world should also result in an impoverished sense of self; a self not embellished with a recognized emotional dimension. In other words, if the interoceptive signals modulated by oxytocin are not contextualized, they will prevent the development of high level representations or generative models of the interoceptive and emotional self—especially a sense of self that is distinct from the ‘other’. The concept of an underdeveloped sense of self appears in several theories of autism including early psychoanalytic theories (Lutz, 1968), Theory of Mind deficits (Baron-Cohen et al., 1985) and weak central coherence (Happe and Frith, 2006), and is consistent with clinical observations in autism; for review, see (Uddin, 2011). Young autistic children do not preferentially orient to the sound and their own name (Osterling and Dawson, 1994) or face (Gunji et al., 2009), exhibit delays in recognizing themselves in a mirror (Dawson and McKissick, 1984, Lind and Bowler, 2009, Spiker and Ricks, 1984) and videos (Dissanayake et al., 2010), and possess diminished self-monitoring skills (Russell and Jarrold, 1999). Autistic adults do not retain detailed biographical memories to the same extent as normal controls (Bruck et al., 2007, Crane and Goddard, 2008) and demonstrate less of a propensity toward introspection (Hurlburt et al., 1994).

During self-evaluation, autistic individuals activate the ventromedial prefrontal cortex and the mid cingulate differently than neurotypical controls (Kennedy and Courchesne, 2008, Lombardo and Baron-Cohen, 2011) and a recent meta-analysis of 24 fMRI studies confirmed that autistic individuals do not engage the right anterior insula to the same extent as neurotypical individuals during social tasks (Di Martino et al., 2009). These imaging studies illuminate the functional anatomy that may underlie the deficits in emotional regulation and ‘sense of self’ observed in autism. The relationship between emotional self-understanding and social cognition has also been supported by the finding that oxytocin improves performance on the Reading the Mind through the Eyes test (RMET) to a greater degree in those individuals who scored higher on a scale of alexithymia (Luminet et al., 2011) and can improve a subjects ability to distinguish self from others (Colonnello et al., 2013).

A failure to elaborate deep generative models of the embodied and socio-emotional self could also explain the coordination difficulties associated with autism (Damasio and Maurer, 1978), in the sense that it would preclude ‘action observation’, could explain their poor attention to biological movement (Kaiser and Pelphrey, 2012) and their action understanding impairments (Wyk et al., 2009). Not only would imitation based learning be disrupted, as described above, but without an appropriate model of the embodied self, less effective and structured proprioceptive predictions (corollary discharge) will be transmitted to premotor regions, impairing their ability to execute complex movements (Muller et al., 2004). A recent study examining the generative (forward) models of action in autism found that the coordination difficulties resulted from a difficulty attenuating proprioceptive information (Haswell et al., 2009). This is a further example of a failure of sensory attenuation in the context of active inference.

### 5.6 Autism and homeostatic regulation

A failure to attenuate interoceptive signals immediately suggests an autonomic hypersensitivity and consequent difficulties in interoceptive and emotional regulation (cf., an ‘autonomic Parkinsonism’). Numerous disturbances in autonomic regulation have been documented in autistic children. Autistic individuals exhibit many symptoms of heightened baseline sympathetic activity and poor autonomic regulation, resulting in less parasympathetic tone (Porges, 1995), as demonstrated by gastrointestinal disturbances (constipation and stomach upset) (Mazefsky et al., 2013, Peeters et al., 2013), reduced respiratory induced sinus arrhythmias of the heart (a measure of vagal or parasympathetic tone) (Bal et al., 2010, Ming et al., 2005, Vaughan Van Hecke et al., 2009), and heightened skin conductance responses (Hirstein et al., 2001). These hyper-reflexic signs are consistent with a failure of oxytocin-dependent attenuation and contextualization of autonomic reflexes.

### 5.7 Autism and self-stimulation

Repetitive self-stimulation behaviors such as scratching, head banging, twirling, and rocking – often labeled as stereotypies – commonly occur in autism (American Psychiatric Association, 2000, Hutt and Hutt, 1968) and may also reflect a failure of sensory attenuation. Kanner's early description of the ‘self-absorbed child’ fits comfortably with the idea that autistic individuals have difficulty disengaging their attention from interoceptive signals. Sensory attenuation during self-made acts normally attenuates their sensory consequences (Brown et al., 2011). However, a failure of sensory attenuation in autism may render the interoceptive consequences of self-stimulation ‘attention grabbing’ and irrepressibly engaging. Despite its social inappropriateness, self-stimulation in autism appears to be rewarding, and difficult to curtail (Boyd et al., 2012), suggesting that these behaviors are not subject to the same degree of sensory attenuation as in neurotypical individuals.

### 5.8 Autism and the stress response

Compromised interoceptive control and reduced interoceptive attenuation could translate into an emotional state resembling anxiety. Autistic individuals report many anxiety symptoms, especially as they mature into adulthood. A recent meta-analysis of comorbidity in the adolescent and young adult population with autism spectrum disorder found that almost 40% suffered from a clinically significant anxiety disorder (van Steensel et al., 2012). Paulus and Stein (Paulus and Stein, 2006) have advanced a predictive coding model for anxiety, founded upon the notion that a large interoceptive prediction error institutes a sympathetic ‘fight-or-flight’ response characteristic of panic. Anxiety symptoms in autism may reflect the failure to attenuate these prediction errors that are thought to be responsible for exaggerated stress responses in normal subjects who are prone to panic (Paulus and Stein, 2006). Here again, we converge on a failure to attenuate interoceptive prediction errors.

### 5.9 Autism and empathy

By its very name, autism connotes self-absorption, and apparent deficits in empathy towards others have long been a presumed hallmark of the disorder (Baron-Cohen et al., 1985, Frith and de Vignemont, 2005, Kanner, 1943). In a predictive coding model, a failure of interoceptive attenuation would impact an individual's ability to exercise both cognitive empathy and compassionate behavior, but would not curtail affective empathy, or its precursor, emotional contagion to the same extent. The relationship between a well-developed sense of self and an ability to infer the mind of others has been noted previously (Gallese, 2003); and studies of empathy confirm that poor self-regulation of emotional responses results in a failure to act empathetically (Decety and Jackson, 2004, Decety and Lamm, 2006). Individuals who have less well-developed ego-boundaries – and merge their identity with another – can experience emotional contagion and affective empathy, but this easily overwhelms them and seldom translates into empathetic or compassionate behavior. As our model predicts, individuals with autism often respond with emotional contagion, but have difficulties translating emotional contagion into cognitive empathy (Blair, 2005, Cox et al., 2012). These findings fit comfortably with difficulties in attenuating autonomic echopraxia, when observing the emotional responses of others. Without appropriate suppression of the affective autonomic reactivity – and a precise understanding of the boundaries between the ‘self’ and ‘others’, Theory of Mind will fail to develop, resulting in the apparently insensitive behavioral responses typical of autism.

## 6 Genetic and epigenetic evidence for a link between autism and oxytocin

Inspired by the social neuroscience findings on oxytocin and behavior, the search for a link between the oxytocin system and autism has gained traction over the past decade. Oxytocin peptide and plasma level abnormalities (Green et al., 2001, Modahl et al., 1998), receptor genetic variant associations (Campbell et al., 2011, Liu et al., 2010, Skuse et al., 2014, Wermter et al., 2010, Wu et al., 2005), and epigenetic alterations (Gregory et al., 2009, Gurrieri and Neri, 2009, Jacob et al., 2007, Kumsta et al., 2013) have been observed in autistic populations, providing support for a connection between atypical oxytocin genetics or epigenetic regulation and autism. The model we present here suggests that in order to initiate the cascade of events that eventually evolve into the autistic phenotype, an early dysfunction in the oxytocin system most likely occurs. As animal work has shown, the fingerprints of such an abnormality could persist well beyond an initial vulnerable period (Bales et al., 2004, Bales and Perkeybile, 2012, Veenema, 2012, Winslow et al., 2003).

## 7 Therapeutic implications

By casting the oxytocin-dependent failure of interoceptive attenuation in a developmental context, a circular dependency emerges between a primary deficit in interoceptive processing and the learning of interpersonal and emotional generative models. The experience-dependent plasticity inherent in this learning will necessarily entail the contextualization of interoceptive and exteroceptive cues that underwrite attention, affordance, and sensory attenuation. A failure to learn this model renders the autistic child insensitive to the contingencies that would normally equip them with descending neuromodulatory control over their sensory signals. One can imagine that the ensuing vicious cycle gives rise to the devastating consequences observed in autism. This circular dependency has implications for therapeutic interventions: exogenous oxytocin in this population alone may not ameliorate symptoms. Instead, administration of oxytocin may need to be combined with behavioral (learning) paradigms to leverage the plasticity unleashed by this neuropeptide.

The current evidence for oxytocin as a therapeutic intervention in autism supports a more nuanced approach. Small clinical studies have suggested that intranasal administration of oxytocin improves social cognition individuals with ASD, both acutely (Andari et al., 2010, Domes et al., 2013, Guastella et al., 2010), and over a six week period (Anagnostou et al., 2012). Several other small clinical studies with oxytocin have also produced some suggestive results in autistic populations (Green and Hollander, 2010, Hollander, 2003, Hollander et al., 2007, Tachibana et al., 2013, Theodosis et al., 2006). However, an open label long-term trial of oxytocin in adolescents with autism over a six month period did not meet the primary outcome measures, although some social reciprocal communication and attention to social stimuli improved modestly (Tachibana et al., 2013). The animal literature predicts that long-term treatment may produce undesirable effects on social behavior, at least in control populations, resulting in indiscriminant social interactions (Huang et al., 2014). These findings suggest that straightforward administration of oxytocin for autistic symptoms may not be optimal. The theory put forward in this review anticipates potential difficulties with chronic administration of oxytocin in autism (after the signs and symptoms have become established) and calls for examining a more targeted (possibly neurodevelopmental) approach that reinforces the proper contextualization needed to assign saliency and promote appropriate models of the prosocial self.

## 8 Limitations of the model and future directions

Although this paper reviews the evidence for a dysfunction in oxytocin mediated interoceptive signal modulation; as it relates to homeostatic regulation, the development of generative models of the self, and sensory attenuation in autism, we have neither attempted to detail how a primary dysfunction in the oxytocin system occurs nor to incorporate the numerous genetic and neurochemical abnormalities associated with autism into this hypothesis. Many abnormalities or associations intersect with the oxytocin system at some level and provide support for our hypothesis. Among the neurotransmitters implicated in autism, anomalies in the serotonin system have been the most closely examined. Demonstrations of abnormal platelet serotonin levels in the autistic population occurred early in the field (Ritvo et al., 1970) and serotonin modifying medications, such as the selective serotonin re-uptake inhibitors, remain one of the most widely prescribed medications for the treatment of autism today (Doyle and McDougle, 2012), (although with mixed efficacy, see Williams et al., 2013). Current evidence suggests that serotonin interacts with the oxytocin system to mediate the contextualization of social cues, a process crucial to normal development and central to the arguments in this review. A recent study demonstrating that social reward requires coincident activation of both postsynaptic serotonin receptors as well as presynaptic oxytocin receptors in the nucleus accumbens (Dolen et al., 2013), provides an important example, where the acquisition of social saliency, a process that becomes vulnerable in autism, depends on oxytocin.

The GABA neurotransmitter system has also been implicated in the pathogenesis of autism, through both genetic linkages with regions coding for GABA-A receptor subunits in families with autism (Ashley-Koch et al., 2006, Collins et al., 2006, Ma et al., 2005, Menold et al., 2001) and as a key component in the postulated inhibition/excitation imbalance theory of autism. As mentioned in the section on perinatal development and oxytocin (Section 4.2), oxytocin profoundly alters GABAergic neurotransmission by diminishing the activity and expression levels of NKCC1, the co-transporter that helps regulate the chloride reversal potential in neurons expressing GABA-A receptors. As an upstream modulator of this co-transporter, oxytocin helps determine inhibitory neurotransmission. From a computational perspective, the oxytocin mediated modulation of inhibitory intrinsic (GABA) connectivity is important. This is because the effective postsynaptic gain of pyramidal cells depends, in large part, on recurrent self-inhibition. This is probably one of the key ways that gain control (and implicit encoding of precision) is implemented in the brain. For example, a large amount of evidence ties together GABAergic neurotransmission, attention and fast synchronous dynamics in visual cortex (Buia and Tiesinga, 2008, Grabenhorst and Rolls, 2010, Johnson et al., 1995, Meinke et al., 2006, Thiel et al., 2009). If oxytocin modulates inhibitory interneurons – that mediate the recurrent inhibition of superficial pyramidal cells – then it is in a position to modulate the precision of prediction errors, from the perspective of predictive coding.

Variants in the vasopressin receptor have also emerged as candidate targets to explain autism (Kim et al., 2002). Because vasopressin frequently operates in opposition to oxytocin (for review see Carter et al., 2008), an overactive vasopressin system could produce many of the same effects that a disrupted oxytocin system triggers. The primary reason for our focus on oxytocin – instead of vasopressin – stems from the known role of oxytocin in the birth process, attachment, and affiliative behaviors. Although vasopressin participates in certain aspects of social behavior (Pitman et al., 1993, Viviani and Stoop, 2008, Wacker and Ludwig, 2012, Walum et al., 2008), it does not have the central role in fetal attachment that lays the foundation for the interoceptive associative learning that we suppose is crucial to developing generative models of the self and for contextualizing exteroceptive socially salient cues—models that then become the basis for appropriate interoceptive attenuation and for developing theory of mind.

Other models of autism, such as the extreme male brain (Baron-Cohen, 2002), suggest that steroid hormones, or other sex specific regulatory genes (Sarachana et al., 2011), might also contribute to the manifestation of this disease. Sex steroids differentially regulate the oxytocin neuropeptide and receptor expression level (Murakami et al., 2011, Tribollet et al., 1990) and this differential modulation could explain why autism afflicts five times more males than females (Chakrabarti and Fombonne, 2001).

We have not discussed possible etiological reasons for a disrupted oxytocin system nor have we addressed why some individuals with autism may experience certain symptom clusters to a greater extent than others. Many different physiological and genetic insults could potentially disrupt the oxytocin system and some of the heterogeneity of phenotypic expression could result from these differences. Polymorphisms in the oxytocin receptor gene (Campbell et al., 2011, Liu et al., 2010, Skuse et al., 2014, Wermter, 2010, Wu et al., 2005), epigenetic methylation of the oxytocin peptide or receptor gene (Gregory et al., 2009, Gurrieri and Neri, 2009, Jacob et al., 2007, Kumsta et al., 2013), genetic variants of CD38, a peptide crucial for oxytocin release (Lerer et al., 2010), and prenatal or perinatal exposures to endocrine disruptors (de Cock et al., 2012), could all intersect with the common downstream impairment of the oxytocin system, but the degree of disruption, the physiological compensatory mechanisms available for a given insult, and the tissue specificity may differ, resulting in heterogeneous phenotypic expression.

The reliability of oxytocin assays has been questioned (Sluss, 2014) and controversies surrounding the correspondence between oxytocin levels in the plasma or saliva and the central nervous system have not yet been resolved (Amico et al., 1983, Kagerbauer et al., 2013), thus limiting the ability to directly test the oxytocin system and its responsivity in large autistic cohorts. Functional brain imaging has partially circumvented this problem by examining the downstream effects of oxytocin on brain activation and connectivity—and could be employed to follow the oxytocin system through development in humans. Additionally, an extension of the Scheele et al. study, using self-induced touch, in addition to the externally applied touch reported in their paper, with and without oxytocin, might elucidate whether oxytocin modulates sensory attenuation during self-generated stimulation as well as influencing the context dependent top-down neuromodulation already demonstrated. Given the converging evidence that symptom expression in autism results from a failure of interoceptive sensory attenuation, proving oxytocin's role in this computational operation could help suggest future therapies and guide possible avenues of research.

## 9 Conclusion

Our Bayesian account of neurodevelopment in autism rests on the role of oxytocin in modulating the precision of interoceptive signals. The implicit contextualization of interoceptive cues underwrites the experience-dependent plasticity necessary to acquire generative models of the emotional self (and others). In this framework, a (primary) developmental pathophysiology in the oxytocin system – and the (secondary) failure to elaborate generative models of the interpersonal world – prevents autistic infants from recognizing and selectively attending to emotionally salient cues. As a result of this oxytocin dependent failure to prescribe appropriate salience to exteroceptive cues and to facilitate interoceptive attenuation, an onslaught of unfiltered sensory information overwhelms the child, promoting protective behaviors and derailing the observational learning so important in early development. Thus, impaired interoceptive inference initiates a cascade of difficulties culminating in a – now all too common – autistic phenotype. As the British scientist Ross Ashby in 1962, stated: “*so, as regulation is the one function that counts biologically, we can expect that natural selection will have made the brain as determinate as possible*”. This simple observation lays the foundation for how a single neuropeptide system could have such specific, yet widespread and devastating effects, especially when considered in a developmental framework.
